# Disseminated Microsporidiosis in an Immunosuppressed Patient

**DOI:** 10.3201/eid1807.120047

**Published:** 2012-07

**Authors:** Eric G. Meissner, John E. Bennett, Yvonne Qvarnstrom, Alexandre da Silva, Emily Y. Chu, Maria Tsokos, Juan Gea-Banacloche

**Affiliations:** National Institutes of Health, Bethesda, Maryland, USA (E.G. Meissner, J.E. Bennett, E.Y. Chu, M. Tsokos, J. Gea-Banacloche);; and Centers for Disease Control and Prevention, Atlanta, Georgia, USA (Y. Qvarnstrom, A. da Silva)

**Keywords:** allogeneic stem cell transplantation, microsporidia, multiple myeloma, disseminated microsporidiosis, fungi, immunosuppressed, *Tubulinosema acridophagus*, novel human pathogen

## Abstract

We report a case of disseminated microsporidiosis in a patient with multiple myeloma who had received an allogeneic stem cell transplant requiring substantial immunosuppression. The causative organism was identified as *Tubulinosema acridophagus,* confirming this genus of microsporidia as a novel human pathogen.

Microsporidia fungi are human pathogens known for causing diarrheal illness in persons infected with HIV; however, there is growing awareness of their involvement in other cases of host immunosuppression. A case of *Tubulinosema* sp. microsporidian myositis was recently reported in a patient with chronic lymphocytic leukemia (*1*). We describe a second case of disseminated microsporidiosis caused by a *Tubulinosema* sp. in an immunosuppressed patient who received an allogeneic stem cell transplant for multiple myeloma.

## Case Report

The patient was a 33-year-old woman with multiple myeloma. After receiving an autologous stem cell transplant (SCT), she experienced a relapse of disease and was enrolled in an experimental protocol of immunoablative chemotherapy, followed by hematopoietic SCT at the National Institutes of Health Clinical Center in Bethesda, Maryland (www.ClinicalTrials.gov identifier NCT00520130). Patients in this study are given a conditioning regimen of chemotherapy drugs (including fludarabine and cyclophosphamide), followed by prophylaxis against graft-versus-host disease (alemtuzumab and cyclosporine).

The patient received a 7/8 HLA-matched allogeneic peripheral blood SCT (with a single mismatch at the DRB1 locus) from an unrelated donor. Her clinical course was complicated by vancomycin-resistant *Enterococcus faecium* bacteremia, meningitis, and concomitant noncommunicating hydrocephalus and retinal hemorrhages. The bone marrow did not reconstitute, and 35 days after the initial transplant, the patient received a second SCT from the same donor after a conditioning regimen with antithymocyte globulin. Engraftment took place on day 49, 14 days after the second transplant. Progressive respiratory failure and pulmonary infiltrates had developed over the preceding week despite administration of broad-spectrum antimicrobial drugs. Results of a bronchoscopy on day 49 showed diffuse alveolar hemorrhage and did not identify a pathogen. Treatment with activated factor 7 and corticosteroids was given with some clinical improvement as well as improvement shown on chest radiograph.

A second bronchoalveolar lavage (BAL), performed on day 64, again showed diffuse alveolar hemorrhage and absence of pathogens. The patient received a second course of corticosteroids and activated factor 7. On day 77, an ophthalmologic examination was performed during a routine follow-up, and new retinal lesions suggestive of candida chorioretinitis were seen. Liposomal amphotericin B was substituted for prophylactic anidulafungin, and an intravitreal injection of amphotericin B was given for a subfoveal lesion.

**Figure 1:**
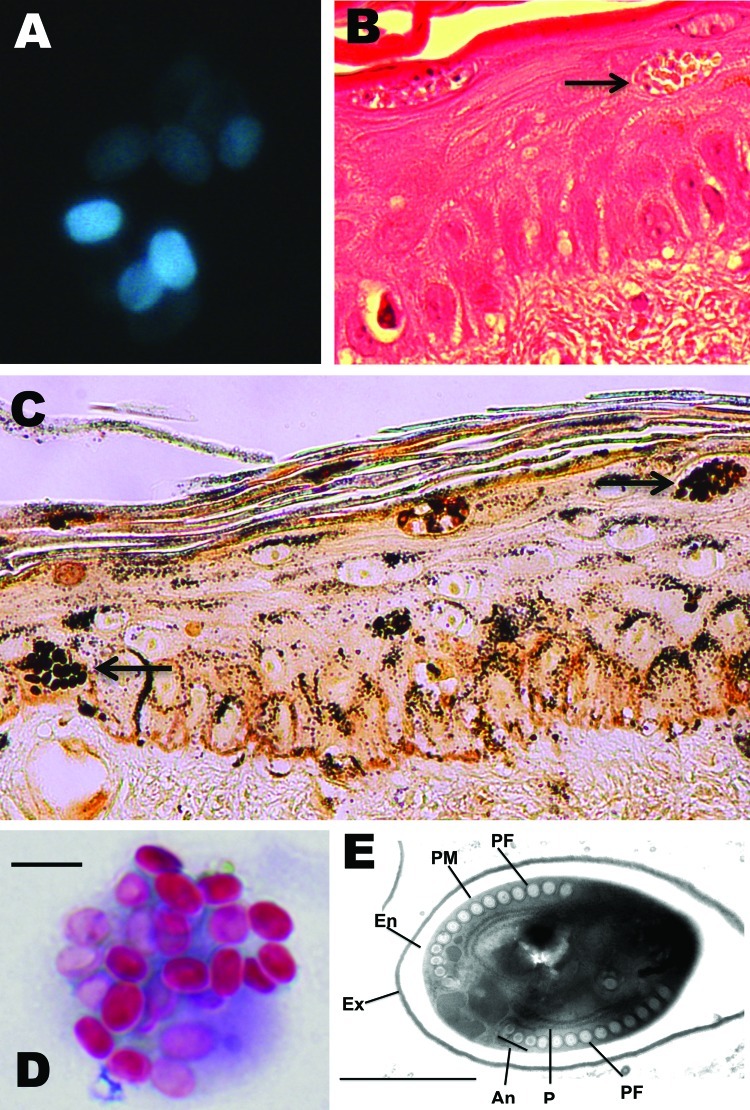
Microsporidium detected in clinical specimens from a stem cell transplant patient who had undergone substantial immunosuppression. A) Calcofluor white–stained ascitic fluid (original magnification ×500). B) Hematoxylin and eosin–stained skin biopsy sample (original magnification ×400). The arrow indicates clusters of spores. C) Warthin-Starry–stained skin biopsy sample (original magnification ×400). The arrows indicate clusters of spores. D) Modified trichrome–stained material from bronchoalveolar lavage. Scare bar = 5.0 μm. E) Transmission electron micrograph depicting 1 of the microsporidian spores identified in a skin biopsy sample. The image shows the polar filament (PF), containing 13 to 14 coils, in a single layer with anisofilar arrangement (An); the plasma membrane (PM); the exospore (Ex); the endospore (En); and polyribosomes (P). Scale bar = 1 μm.

**Figure A1:**
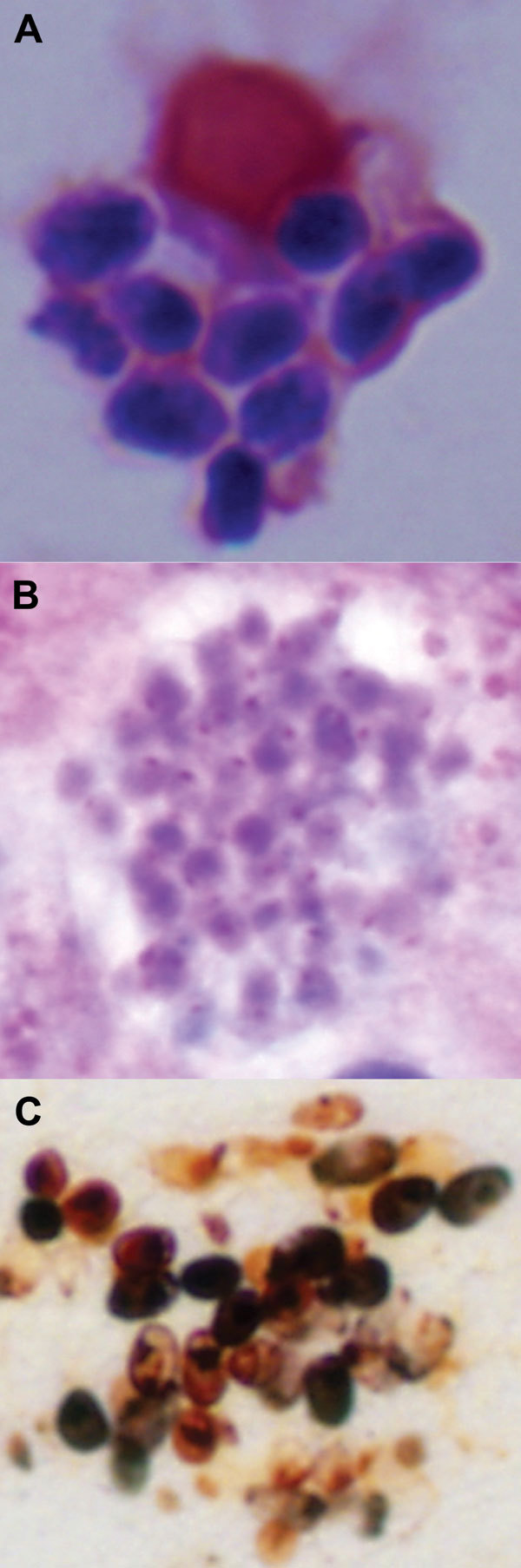
Microsporidium detected in clinical specimens from a stem cell transplant patient who had undergone substantial immunosuppression. A) Gram-stained ascitic fluid (original magnification 1,000×). B) Hematoxylin and eosin–stained liver biopsy sample. C) Warthin-Starry–stained liver biopsy sample (original magnification 600×).

At this time, the patient also had increasing hyperbilirubinemia and elevation of liver aminotransferases, together with diarrhea, abdominal distension, and new ascites. Graft-versus-host disease of the gut and liver was suspected. A colonoscopy on day 79 showed that, with the exception of 1 ulcer, the colonic mucosa appeared normal; biopsy samples showed nonspecific inflammation and a few apoptotic bodies. A liver biopsy and paracentesis were performed on day 85. Samples were stained with calcofluor white, which revealed yeast-like organisms 2–3 μm in diameter (Figure 1, panel A; Figure A1, panels A and B). The samples were also cultured for the presence of fungi, but results were negative.

Over the next 10 days, despite treatment with broad-spectrum antimicrobial drugs and corticosteroids, the patient had intermittent fever, continued elevation of liver enzymes, and progressive respiratory insufficiency with episodes of diffuse alveolar hemorrhage. Examination of BAL samples on days 90, 92, 96, and 97 was unrevealing. On day 96, multiple discrete, nonblanchable red macules and papules developed on the patient’s face, arms, legs, and trunk; the macules and papules were initially suggestive of disseminated candidiasis. Skin biopsy showed cysts mostly within the epidermis and follicular epithelium, similar to those seen previously in the liver biopsy sample and in ascitic fluid; these cysts featured closely packed, uniform, oval basophilic structures (Figure 1, panels B and C).

On day 96, diffuse alveolar hemorrhage recurred. BAL was performed, and staining with Diff-Quik (Siemens Healthcare Diagnostics Inc., Deerfield, IL, USA) and modified trichrome showed that the fluid samples were now positive for intracellular yeast-like forms. In retrospect, we determined that the BAL samples from day 90 also contained yeast-like forms, but they could not be identified at that time (Figure 1, panel D). Refractory hypoxemia developed, and the patient died on day 97 after the initial transplant. No postmortem examination was performed.

The remaining clinical specimens were subsequently examined to determine if the yeast-like forms were microsporidia. Warthin-Starry staining showed that all organisms in the skin and ascitic fluid were consistent with microsporidia (Figure 1, panel C; Figure A1, panel C). Similar organisms were identified in the BAL samples from days 90 and 96; spore size averaged 3.5 μm (range 3.3–3.9 μm) (Figure 1, panel D). Results of calcofluor white and modified acid-fast staining of the organisms were also positive, consistent with microsporidia.

Electron micrographs of skin and peritoneal fluid revealed microsporidian spores with features that were compatible with the genus *Tubulinosema*. Spores had a single layer of polar tubule or filament with 11–14 coils; the last 3 coils were clearly smaller in some spores (i.e., they had an anisofilar polar tubular arrangement) (Figure 1, panel E). The quality of the electron micrograph was limiting and did not allow visualization of the diplokaryon nucleus.

**Figure 2:**
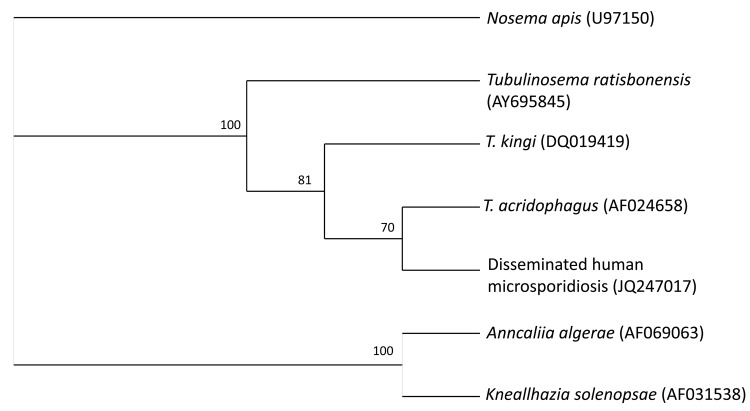
Cladogram of *Tubulinosematidae* spp. based on small subunit ribosomal RNA gene sequences. *Nosema apis* was added as an outgroup. The phylogenetic tree was created by using the quartet puzzling maximum likelihood program TREE-PUZZLE (www.tree-puzzle.de). The numbers at the nodes are quartet puzzling estimations of support to each internal branch.

BAL samples were sent to the reference diagnostic laboratory for parasitology at the Centers for Disease Control and Prevention for molecular analysis. A nested PCR approach was applied, using the outer primers microFN18 (5′-CACCAGGTTGATTCTGCC-3′) and microR9 (5′-GTATGATCCTGCCACAGATTCTTCTAT-3′) and the inner primers Ta50f (5′-GGTTGATTCTGCCTGTTATATGT-3′) and Ta1300r (5′-GGACACATTCATCGTAACTTAGT-3′). Phylogenetic analysis of the resulting 1,246-bp sequence (GenBank accession no. JQ247017) revealed a high similarity to the small subunit ribosomal RNA gene from *T. acridophagus* (Figure 2), with only 1 nucleotide difference between the 2 sequences. Our final clinical diagnosis was disseminated *T. acridophagus* microsporidiosis with skin, liver, peritoneal, lung, and possibly chorioretinal involvement. Histopathology was notable for a paucity of inflammation around the organisms.

## Conclusions

Along with another case of *Tubulinosema* sp. microsporidiosis (*1*), this case of disseminated microsporidiosis in an immunosuppressed SCT patient solidifies *Tubulinosema* sp. as a novel pathogen in immunosuppressed persons. By using molecular analysis, we characterized the microsporidian from the patient in our study to the species level (*T. acridophagus*).

Of 3 other reported cases of pulmonary microsporidiosis after SCT, 2 were in T cell–depleted patients (*2**,**3*), and the third was in a patient receiving high-dose methylprednisolone (1 g/m^2^) to treat graft-versus-host disease (*4*). The patient in our study was substantially immunosuppressed. She received alemtuzumab with her first allogeneic transplant and conditioning with antithymocyte globulin before her second transplant, resulting in significant in vivo T-cell depletion; she was also receiving adrenal corticosteroids. Because the patient had retinal changes suggestive of candidemia, we initially suspected that she had a disseminated infection. Although we did not identify the cause of these retinal changes, a case of endopthalmitis caused by microsporidia has been reported (*5*).

Microsporidia have only rarely been reported in the skin (*6**–**8*). The organisms we found in the skin and liver of the patient in our study were similar, providing the clue to the eventual postmortem diagnosis of disseminated microsporidiosis. This report reinforces the need to consider microsporidiosis as a life-threatening condition and a potential cause of diffuse alveolar hemorrhage after SCT (*2**,**3*), particularly in patients with extreme T-cell dysfunction.
